# Intravenous iron and erythropoietin therapy for postoperative anemia among orthopedic surgery patients

**DOI:** 10.1186/s13018-023-03926-y

**Published:** 2023-07-18

**Authors:** Huixin Chen, Jing Yu, Qing Wei, Yu Zhang, Xilin Ouyang, Shun Wang

**Affiliations:** 1 Department of Blood Transfusion, Wuhan Hospital of Traditional Chinese and Western Medicine, Tongji Medical College, Huazhong University of Science and Technology, Wuhan, China; 2 Department of Transfusion, Tongji Hospital, Tongji Medical College, Huazhong University of Science and Technology, Wuhan, China; 3 Department of Blood Transfusion, Chinese PLA General Hospital Fourth Medical Center, Beijing, 100048 China; 4 Department of Clinical Laboratory, Wuhan Hospital of Traditional Chinese and Western Medicine, Tongji Medical College, Huazhong University of Science and Technology, Wuhan, 430022 China

**Keywords:** Postoperative anemia, Erythropoiesis, Iron metabolism, Recombinant human erythropoietin, Intravenous iron

## Abstract

**Background:**

Postoperative anemia is a risk factor for adverse surgical outcomes. Our study aimed to assess the role of intravenous iron and erythropoietin therapy for the rapid correction of anemia following orthopedic surgery.

**Methods:**

Patients undergoing elective orthopedic surgery were prospectively enrolled and randomly divided into three groups: Control (placebo), Group 1 (IV iron monotherapy), and Group 2 [combined IV iron and recombinant human erythropoietin (rHuEPO) therapy]. Blood tests were performed preoperative (baseline) and on postoperative days (PODs) 1, 3, and 7.

**Results:**

All groups demonstrated significantly lower hemoglobin (Hb) concentrations compared to baseline, with no significant inter-group differences in postoperative Hb concentrations (*p* > 0.05). Serum erythropoietin, ferritin, and vitamin B_12_ levels, and reticulocyte count increased beyond normal ranges in all groups. Significantly lower serum iron levels were observed postoperatively in all groups (*p* < 0.05). No significant inter-group differences in hepcidin level were observed (*p* > 0.05).

**Conclusion:**

Postoperative treatment with combined intravenous iron and rHuEPO was ineffective in correcting postoperative anemia among orthopedic surgery patients, besides achieving higher reticulocyte counts in the first week of surgery. No improvement in mobilization of storage iron was achieved with rHuEPO. We further suggest against vitamin B_12_ administration during the early postoperative period.

## Background

Postoperative anemia represents a common complication of major surgeries and is a risk factor for adverse surgical outcomes [1]. Increasing attention is being paid to the management of this complication. Postoperative anemia is mainly attributable to perioperative blood loss, including those secondary to surgical bleeding, coagulopathies, and phlebotomies, as well as inflammation-induced blunted erythropoiesis [1]. Evidence has shown its closer resemblance to anemia of chronic disease (ACD) rather than iron deficiency anemia [2]. Besides RBC transfusion, other treatment options for postoperative anemia may include dietary iron supplementation, intravenous (IV) iron therapy, and recombinant human erythropoietin (rHuEPO) therapy. While the effectiveness of dietary iron replacement is, at best, minimal, the latter two approaches have demonstrated a role in the management of other anemia types with similar pathophysiological features to postoperative anemia, such as anemia of chronic diseases [3, 4]. In addition, a standardized treatment strategy for postoperative anemia, specifically, does not currently exist. The effects of IV iron therapy on the early recovery of postoperative anemia remain uncertain [5–7]. Moreover, very few randomized controlled trials have demonstrated the ability of rHuEPO in promoting iron utilization and improving postoperative anemia in the early stages.

Our study therefore aimed to evaluate the outcomes of IV iron monotherapy and as combination therapy with rHuEPO for the early correction of postoperative anemia following orthopedic surgery, to better our understanding of the relationship between erythropoietin, iron metabolism, and erythropoiesis.

## Materials and methods

### Patients and treatment

Patients undergoing orthopedic surgery between May 2021 and May 2022 were prospectively analyzed. The inclusion criteria were as follows: age ≥ 18 years and elective orthopedic surgery. The exclusion criteria were as follows: hemodynamic instability requiring rescue therapy; anticoagulant use, including aspirin, clopidogrel, and warfarin; preoperative anemia (baseline hemoglobin levels < 12 g/dL); history of chronic inflammatory disease; evidence of postoperative infection or hemolysis; need for autologous or allogeneic blood transfusion; and refusal to participate in this study.

All participants were randomly assigned to three groups-Group 1 (iron monotherapy), 200 mg IV iron sucrose (Veloft, Vifor Inc., Biologika GmbH, Switzerland); Group 2 (combined therapy), 200 mg IV iron sucrose and 5000 U subcutaneous rHuEPO (Sinovac Biotech Ltd., Ji Nan, China); and Control (placebo), 200 mL normal saline (placebo). All assigned therapies were administered total 3 times on postoperative days (POD) 1, 3, and 7. Iron sucrose was diluted in 200 mL normal saline and was administered over 1 h. All patients were blinded to the assigned IV solutions. Treatment adherence and side effects were closely monitored by a research nurse.

### Measured parameters

Blood tests were performed on the day before the surgery (baseline) and on PODs 1, 3, and 7. Hemoglobin (Hb) concentration and reticulocyte count were measured using the E-5000 Hematology Analyzer (Sysmex TOA, Kobe, Japan). Serum erythropoietin, folate, and vitamin B_12_ levels were measured using the Immulite 1000 chemiluminescent assay (Siemens, Surrey, UK; normal ranges, 5.4-31 mIU/mL, 9.53-45.17 nmol/L, and 177.1-664.2 pmol/L, respectively). Iron metabolism was analyzed in terms of serum iron level (normal range 9-30 µmol/L), transferrin level (normal range 2-3.6 g/L), transferrin saturation (TSAT) (calculated from serum iron and transferrin concentration; normal range 25.1-51.9%), and ferritin level (normal range 13-400 ng/mL). Serum hepcidin was measured using ELISA (DHP250; R&D Systems, Minneapolis, USA).

### Statistical analysis

All statistical analyses were performed using SPSS 25.0 (IBM Corp., Armonk, NY, USA). Distribution normality was assessed using the Shapiro–Wilk test. Continuous variables were compared using the Mann–Whitney *U* test. The Spearman rank correlation test was used to analyze the associations between variables. The dynamic association of variables was evaluated by days after the operation using the independent *t*-test. *p* values < 0.05 were regarded as statistical significance.

## Results

### Demographic and clinical characteristics

A total of 89 patients were included in our study, of whom 42 (47.19%) and 47 (52.81%) were male and female, respectively. The mean age was 53.2 years (range 24-70 years). Groups 1 and 2 each consisted of 30 patients, while Control included 29 patients. All groups were comparable in age and gender (*p* > 0.05). Surgery types were similar as well (*p* > 0.05). The baseline demographic, surgical, and hematological data of all three groups are summarized in Table 1.

**Table 1:** Baseline demographic and clinical characteristics of the three groups

Characteristics	Group 1 (n = 30)	Group 2 (n = 30)	Control (n = 29)	*p* value
Gender				
Female	19 (63.33%)	12 (40%)	16 (55.17%)	
Male	11 (36.67%)	18 (60%)	13 (44.83%)	
Age (year)	52.6 (27–70)	53.5 (25–70)	52.8 (24–70)	
Type of surgery				
Arthroplasty	5	7	8	
Total hip replacement	8	9	6	
Total knee replacement	9	8	9	
Lumbar laminectomy	9	6	6	
Hb (g/L)	140 (120–159)	127 (120–139)	132 (120–146)	0.065*, 0.073^&^, 0.056^#^
Reticulocyte count	0.058 ± 0.005	0.049 ± 0.007	0.042 ± 0.003	0.067*, 0.073^&^, 0.231^#^
CRP (mg/L)	3.11 (3.01–6.49)	3.53 (2.78–5.82)	3.26 (2.73–6.31)	0.083*, 0.062^&^, 0.143^#^
Intraoperative blood loss (mL)	628 (400–1000)	743 (200–1200)	659 (200–1500)	0.086*, 0.073^&^, 0.052^#^
Postoperative blood loss (mL)	64 (50–150)	71 (50–150)	59 (50–150)	0.354*, 0.421^&^, 0.653^#^

Treatment regimens: Group 1, IV iron monotherapy; Group 2, combined IV iron and rHuEPO therapy; and Control, placebo

*Comparison between Group 1 and Control

^&^Comparison between Group 2 and Control

^#^Comparison between Group 1 and Group 2

No side effects related to IV iron or rHuEPO, including hypersensitivity reactions, hypertension, increased venous thromboembolism risk, and stroke [8], were observed throughout the study period.

### Hb concentration

**Fig. 1:**
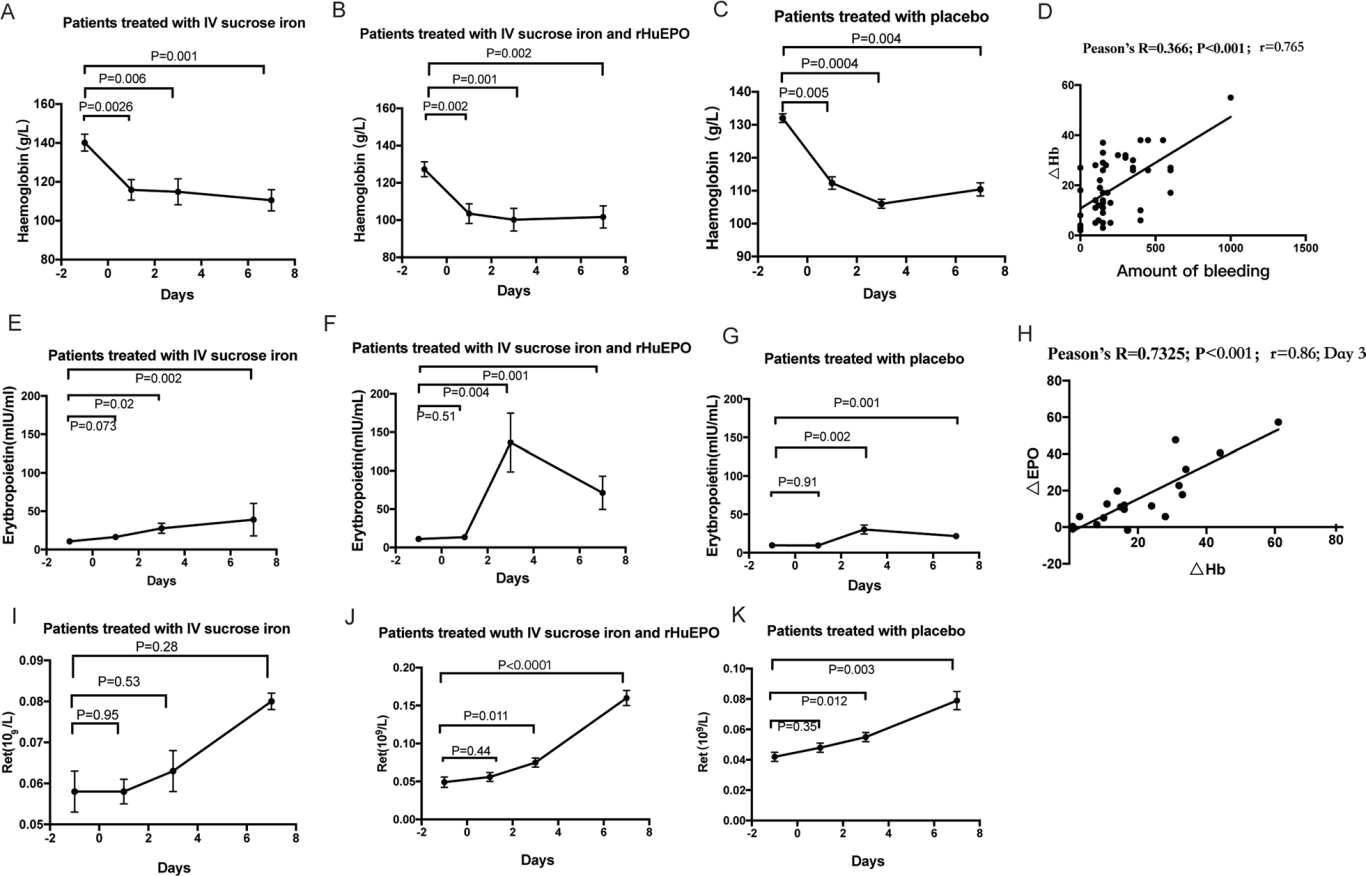
Changes in hemoglobin concentration following IV iron monotherapy (Group 1, **A**), combined IV iron and rHuEPO therapy (Group 2, **B**), and placebo (Control, **C**). The association between the amount of intraoperative blood loss and the drop in Hb concentration on POD1 **D**). Changes in serum erythropoietin following IV iron monotherapy (Group 1, **E**), combined IV iron and rHuEPO therapy (Group 2, **F**), and placebo (Control, **G**). The association between serum erythropoietin and Hb concentration (**H**). Changes in reticulocyte count following IV iron monotherapy (Group 1, **I**), combined IV iron and rHuEPO therapy (Group 2, **J**), and placebo (Control, **K**). Δ Hb was defined as the difference in Hb concentration between baseline and POD1. Δ EPO and Δ Hb were defined as the difference in erythropoietin and Hb, respectively, between baseline and POD3

Despite treatment, postoperative anemia was observed in all patients (100%), with significantly lower Hb concentrations compared to normal range observed on POD7 in all three groups (*p* = 0.001, 0.002, and 0.004, respectively; shown in Fig. 1A –C). However, no significant inter-group differences in Hb concentrations were seen at all time points (*p* > 0.05, shown in Table 2). Significant correlation between intraoperative blood loss volume and the decrease in Hb concentration was observed on POD1 (*r* = 0.765, *p* < 0.01, shown in Fig. 1D).

**Table 2:** Changes in hemoglobin concentration (g/L)

Groups	Baseline	POD1	POD3	POD7
Group 1	140.2 ± 4.37	115.9 ± 5.27	114.9 ± 6.68	110.6 ± 5.5
Group 2	127.3 ± 4.01	103.5 ± 5.27	100.2 ± 6.133	101.7 ± 5.91
Control	132 ± 3.66	112.3 ± 5.28	106 ± 5.16	110.4 ± 5.78
*p*1 value	0.16	0.65	0.3	1
*p*2 value	0.39	0.26	0.47	0.31
*p*3 value	0.053	0.12	0.12	0.31

Treatment regimens: Group 1, IV iron monotherapy; Group 2, combined IV iron and rHuEPO; and Control, placebo.

POD, postoperative day; *p*1, Group 1 versus Control; *p*2, Group 2 versus Control; *p*3, Group 1 versus Group 2

All values are expressed as mean ± standard deviation

### Reticulocyte count

Rapid increase in reticulocyte count from baseline was observed in all groups, with significantly higher reticulocyte count observed on POD7 (*p* < 0.0001, = 0.28, and < 0.03, respectively; shown in Fig. 1I–K). Group 2 associated with significantly higher reticulocyte count on POD7 compared to the other two groups (*p* = 0.0001 and 0.0004, respectively; shown in Table 3).

**Table 3:** Changes of reticulocyte count (× 10^12^/L)

Groups	Baseline	POD1	POD3	POD7
Group 1	0.058 ± 0.005	0.058 ± 0.003	0.063 ± 0.005	0.08 ± 0.014
Group 2	0.049 ± 0.007	0.056 ± 0.005	0.075 ± 0.006	0.157 ± 0.01
Control	0.042 ± 0.003	0.048 ± 0.003	0.055 ± 0.002	0.079 ± 0.006
*p*1 value	0.031	0.031	0.13	0.91
*p*2 value	1	0.17	0.003*	0.0001*
*p*3 value	0.41	0.77	0.12	0.0004*

Treatment regimens: Group 1, IV iron monotherapy; Group 2, combined IV iron and rHuEPO; and Control, placebo

POD, postoperative day; *p*1, Group 1 versus Control; *p*2, Group 2 versus Control; *p*3, Group 1 versus Group 2

*p < 0.05. All values are expressed as mean ± standard deviation

### Serum erythropoietin

Significant increase in serum erythropoietin concentrations compared to baseline was observed on POD7 in all three groups (*p* < 0.005, shown in Fig. 1E–H). Group 2 associated with significantly higher serum erythropoietin concentrations on POD3 compared to Control and Group 1 (*p* = 0.03 and 0.02, respectively; shown in Table 4). Significant correlation was observed between the decrease in Hb concentration and the increase in erythropoietin concentration in the Control group (*r* = 0.86, *p* < 0.001, shown in Fig. 1H).

**Table 4:** Changes in serum erythropoietin concentration (mIU/mL)

Groups	Baseline	POD1	POD3	POD7
Group 1	10.68 ± 1.19	16.41 ± 2.7	27.78 ± 6.46	39.16 ± 21.23
Group 2	11.01 ± 3.1	13.43 ± 1.84	136.6 ± 38.21	71.28 ± 21.77
Control	9.66 ± 1.24	9.43 ± 1.56	30.26 ± 5.87	21.6 ± 3.07
*p*1 value	0.58	0.03*	0.59	0.35
*p*2 value	0.67	0.11	0.03*	0.02*
*p*3 value	0.71	0.36	0.02*	0.33

Treatment regimens: Group 1, IV iron monotherapy; Group 2, combined IV iron and rHuEPO; and Control, placebo

POD, postoperative day; *p*1, Group 1 versus Control; *p*2, Group 2 versus Control; *p*3, Group 1 versus Group 2

*p < 0.05. All values are expressed as mean ± standard deviation. Normal range of serum erythropoietin: 5.4–31 mIU/Ml

### Iron metabolism

**Fig. 2:**
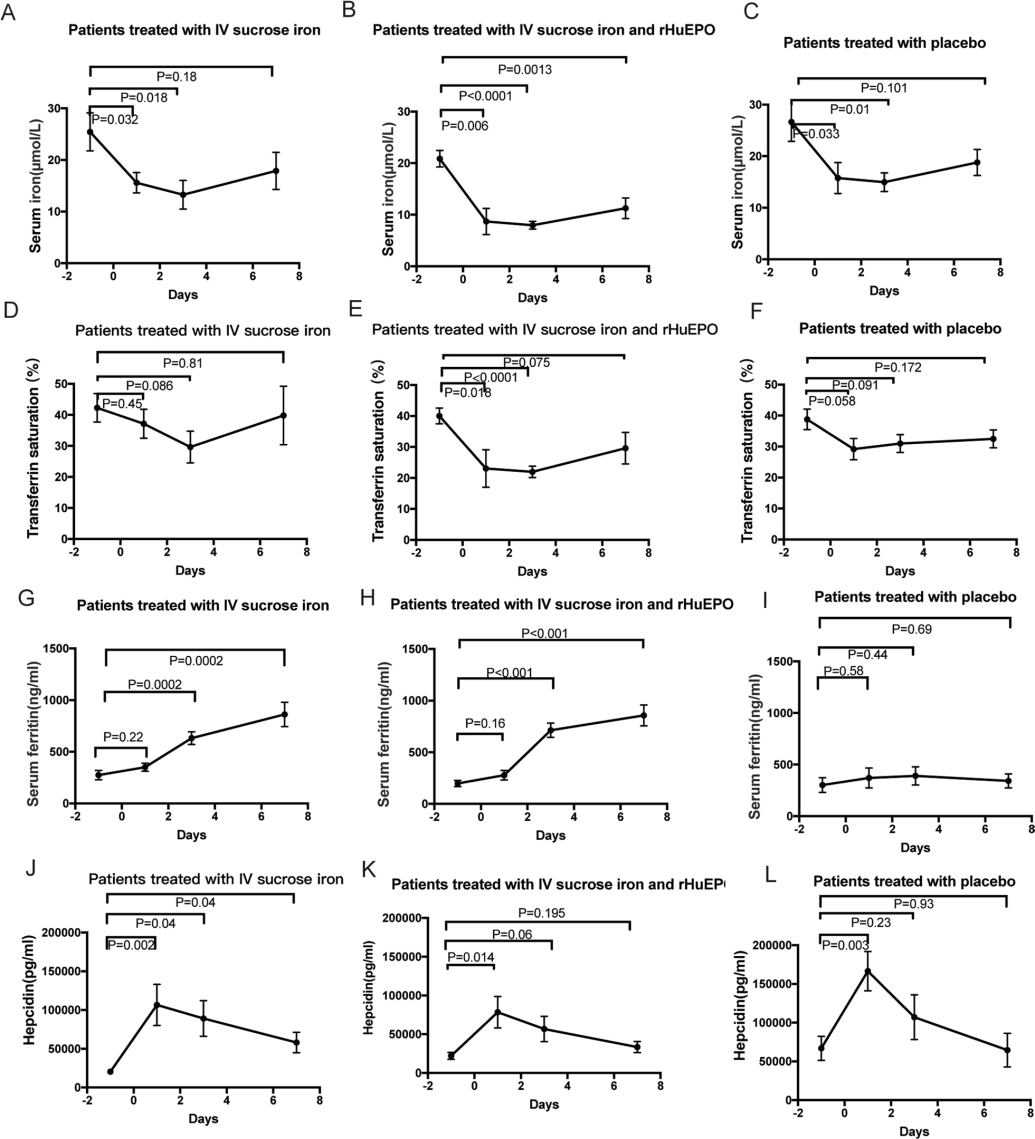
Changes in iron metabolism parameters. Serum iron levels following IV iron monotherapy (Group 1, **A**), combined IV iron and rHuEPO therapy (Group 2, **B**), and placebo (Control, **C**). Serum transferrin saturation following IV iron monotherapy (Group 1, **D**), following combined IV iron and rHuEPO therapy (Group 2, **E**), and placebo (Control, **F**). Serum ferritin levels following IV iron monotherapy (Group 1, **G**), combined IV iron and rHuEPO therapy (Group 2, **H**), and placebo (Control, **I**). Serum hepcidin levels following IV iron monotherapy (Group 1, **J**), combined IV iron and rHuEPO therapy (Group 2, **K**), and placebo (Control, **L**)

Serum iron and TSAT were significantly lower in Group 2 compared to Control and Group 1 on POD3 (9.23 ± 1.28 μmol/L vs. 14.97 ± 1.82 μmol/L, *p* = 0.024; and 21.96 ± 1.84% vs. 30.98 ± 2.89%, *p* = 0.02, respectively; shown in Fig. 2B, E). Significantly higher ferritin levels were demonstrated in Groups 1 and 2 compared to Control on POD7 (861.3 ± 118.4 ng/mL vs. 341.9 ± 68.09 ng/mL, *p* = 0.001; and 857.3 ± 101.3 ng/mL vs. 341.9 ± 68.09 ng/mL, *p* = 0.001, respectively; shown in Fig. 2G, H). Serum hepcidin levels peaked on POD1 in all three groups, at significantly higher levels compared to baseline (*p* = 0.002, 0.014, and 0.003, respectively; shown in Fig. 2J–L). No significant inter-group differences in hepcidin levels were observed at all timepoints (*p* > 0.05, shown in Table 5).

**Table 5:** Iron metabolism and erythropoiesis parameters

	Baseline	POD1	POD3	POD7
*Control*				
Iron (μmol/L)	26.63 ± 3.73	15.75 ± 3.02	14.97 ± 1.82	18.78 ± 2.53
TSAT (%)	38.75 ± 3.33	29.2 ± 3.45	30.98 ± 2.89	32.49 ± 2.88
Ferritin (ng/ml)	302.1 ± 71.87	370.2 ± 97.15	390.7 ± 87.02	341.9 ± 68.09
Hepcidin (pg/ml)	66,898 ± 15,507	166,589 ± 25,313	107,156 ± 28,831	64,595 ± 21,636
Folate (nmol/L)	19.2 ± 2.22	14.25 ± 2.29	13.64 ± 2.57	16.37 ± 1.89
VitaminB_12_ (pmol/L)	1067 ± 152	909.2 ± 162	1191 ± 150.8	1075 ± 171.3
*Group 1*				
Iron (μmol/L)	21.03 ± 2.09	12.93 ± 3.08	11.45 ± 2.56	14.06 ± 3.6
TSAT (%)	42.26 ± 4.58	37.13 ± 4.7	29.63 ± 5.15	39.81 ± 9.43
Ferritin (ng/ml)	273.8 ± 45.44	351.1 ± 39.58	631.9 ± 61.25	861.3 ± 118.4
Hepcidin (pg/ml)	21,937 ± 3951	112,552 ± 24,534	71,852 ± 21,927	54,167 ± 15,376
Folate (nmol/L)	12.54 ± 2.38	7.92 ± 1.58	8.1 ± 0.81	10.02 ± 2.02
VitaminB_12_ (pmol/L)	452 ± 132.4	1070 ± 204.3	1189 ± 190.5	1266 ± 210
*Group 2*				
Iron (μmol/L)	24.79 ± 3.16	10.65 ± 1.94	9.23 ± 1.28	13.99 ± 2.24
TSAT (%)	40.04 ± 2.57	23.07 ± 6.04	21.96 ± 1.84	29.62 ± 5.08
Ferritin (ng/ml)	198.2 ± 30.47	278.2 ± 45.86	713.3 ± 69.14	857.3 ± 101.3
Hepcidin (pg/ml)	22,021 ± 4595	78,443 ± 20,413	56,820 ± 16,460	33,337 ± 7249
Folate (nmol/L)	16.46 ± 1.77	12.29 ± 2.04	11.4 ± 1.52	10.87 ± 1.45
VitaminB_12_ (pmol/L)	1114 ± 169	1356 ± 111.2	1365 ± 111.5	1348 ± 128.1

Treatment regimens: Group 1, IV iron monotherapy; Group 2, combined IV iron and rHuEPO; and Control, placebo

POD, postoperative day; *p*1, Group 1 versus Control; *p*2, Group 2 versus Control; *p*3, Group 1 versus Group 2; all values are expressed as mean ± standard deviation

### Changes in folate and vitamin B_12_

**Fig. 3:**
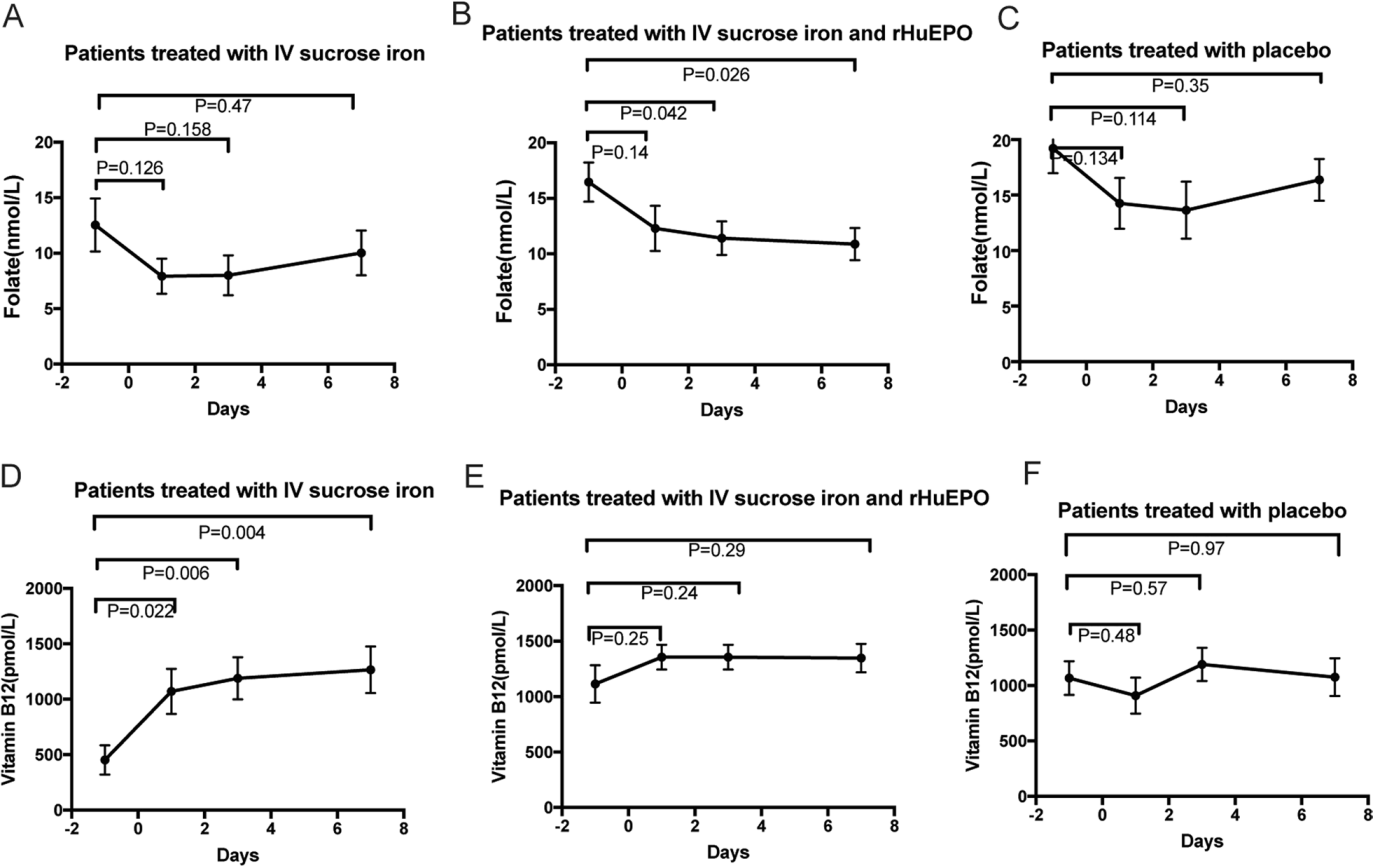
Changes in folate and vitamin B_12_ levels. Serum folate levels following IV iron monotherapy (Group 1, **A**), combined IV iron and rHuEPO therapy (Group 2, **B**), and placebo (Control, **C**). Serum vitamin B_12_ levels following IV iron monotherapy (Group 1, **D**), combined IV iron and rHuEPO therapy (Group 2, **E**), and placebo (Control, **F**)

Significant decrease in serum folate concentration from baseline was observed on POD3 and POD7 in Groups 1 and 2 (*p* < 0.05, shown in Fig. 3B). Serum vitamin B_12_ concentration increased above normal range in all three groups (shown in Fig. 3D–F), with significant increase from baseline observed at all time points in Group 1 only (*p* = 0.022, 0.006, and 0.004, respectively; shown in Fig. 3D). No significant differences in serum folate or vitamin B_12_ concentration were observed between the three groups (*p* > 0.05, shown in Table 5).

## Discussion

Inflammatory responses to surgery may create a relative iron- and erythropoietin-deficient postoperative anemia state that may delay patient recovery from surgery [9]. Perioperative hemorrhage and blunted erythropoiesis secondary to decreased iron availability, with concomitant normal or near-normal erythropoietin levels, have been reported as the two major etiologies of perioperative anemia [10]. Erythropoietin plays an important role in erythropoiesis—the builder of red blood cells; in contrast, iron is a substrate for hemoglobin—the building block of red blood cells. Based on this theory, intravenous iron increases the effectiveness of erythropoiesis-stimulating agents by supplying the builder with sufficient building blocks [11]. Combined therapy therefore carries the potential for the effective correction of postoperative anemia [2].

Significant increase in erythropoietin was observed in all three groups in our study during the first week of surgery, especially following combined IV iron and rHuEPO therapy. While erythropoietin levels remained within normal range among controls, increased levels beyond normal ranges were observed following iron monotherapy and combined therapy. This led to our focus on the effects of exogenous erythropoietin on iron metabolism.

Transferrin saturation (TSAT) is an important biomarker for iron availability, with TSATs < 20% and > 40% correlating with iron deficiency and overload, respectively [12, 13]. Our results showed a significant trough in serum iron and TSAT in all groups on POD3, but a significant increase in serum ferritin on POD7. Furthermore, significantly lower serum iron and TSAT were observed with combined iron and rHuEPO therapy, reflecting transfer of iron from circulation pools into synthesis of intracellular hemoglobin. However, maybe in combined iron and rHuEPO therapy group much of the administered iron was transported into the reticuloendothelial system as storage iron, where it was less readily available for erythropoiesis. Our findings imply that the administration of intravenous iron and rHuEPO in the first week after surgery merely promoted serum iron utilization, but not mobilization of storage iron.

Hepcidin, a 25-amino acid peptide predominantly synthesized by liver cells, regulates iron absorption and recycling by inducing ferroprotein internalization and degradation [14]. Surgery-induced inflammation associates with the upregulation of hepcidin, which has been shown to suppress/hinder erythropoiesis by blocking intestinal iron absorption. Intracellular iron therapy has been reported to partly overcome hepcidin blockade, allowing for iron export into the plasma, and subsequent transport into the bone marrow as transferrin-bound iron for erythropoiesis [15]. Moreover, the administration of rHuEPO may cause hepcidin down-regulation within 24 h [16]. In our study, a significant peak in hepcidin level was observed on POD1 in all groups; however, no significant inter-group differences were seen throughout the postoperative period. This suggests the insignificant role of both iron and rHuEPO supplementation in reducing hepcidin levels and preventing postoperative functional iron deficiency. Our findings were, however, in contradiction to those of previous studies [17, 18].

Postoperative anemia was observed in all of our patients following elective orthopedic surgery. Combined iron and rHuEPO therapy during the first week of surgery did not demonstrate a role in correcting postoperative anemia, although it significantly associated with higher reticulocyte counts compared to iron monotherapy and control.

Hemodilution due to volume overload represents a potential cause for low hemoglobin levels. Hb concentration alone is therefore insufficient for a diagnosis of postoperative anemia, given its influence by plasma volume derangements, which may result in overdiagnosis [19]. In cases of uncomplicated recovery from surgery, a nadir in Hb concentration is mostly observed within the first 3–4 days [1]. As reticulocytes are normally released into the circulation 18–36 h before their final maturation into erythrocytes, their levels provide a real-time assessment of the functional state of erythropoiesis [20]. Our findings of significantly higher reticulocyte levels with combined IV iron and rHuEPO therapy thereby suggest its efficacy in inducing erythropoiesis, but not Hb recovery, in the first week of surgery. This corroborates with the outcomes reported by other similar studies [18, 21], although this was also in contrary to others [22].

Folate, vitamin B_12_, and iron play crucial roles in erythropoiesis. Erythroblasts require folate and vitamin B_12_ for proliferation during differentiation. Deficiency in folate or vitamin B_12_ inhibits purine and thymidylate syntheses, impairs DNA synthesis, and causes erythroblast apoptosis, resulting in anemia secondary to ineffective erythropoiesis [23]. Serum folate concentration is known as the most sensitive biochemical index for folate deficiency [24]. Body folate stores last three to six months, whereas vitamin B_12_ stores last three to six years. In our study, serum folate concentrations were abnormally low following iron monotherapy and combined therapy, but were normal among controls. We therefore suggest the administration of folate as a supplement to IV iron and rHuEPO therapy within the first week of surgery.

Serum vitamin B_12_ concentration has been shown to associate with thrombosis-associated systemic inflammation [25, 26] and poor prognosis among critically ill patients [27]. Our results showed increased postoperative serum vitamin B_12_ concentration from baseline in all three groups; although statistically insignificant, this may have reflected the natural development of postoperative inflammation. We therefore suggest against the administration of vitamin B_12_ within the first week of surgery.

Our study had several limitations. Firstly, the small sample size challenges the generalizability of our results. Although the actual incidence of severe postoperative anemia was high, only 4% were deemed eligible for the study, with the primary reasons for exclusion being the presence of preoperative anemia and the development of postoperative organ dysfunction. Such strict inclusion criteria was implemented to limit the study population to those who would most likely respond to the intervention. Secondly, preoperative Hb concentration and reticulocyte count were randomly obtained within the week prior to surgery and may thus be an inaccurate reflection of their levels at the time of surgery. Thirdly, the follow-up period was short. The 1-week study period was selected based on our hypothesis that any benefits of accelerated recovery from postoperative anemia, such as reduced RBC transfusion and length of hospitalization, would most likely be observed within that time period in real clinical practice. Fourthly, occult blood loss was not evaluated in our study, due to the difficulties in defining its real impact on total blood loss.

## Conclusions

In summary, our prospective, double-blinded, randomized controlled trial demonstrated that early treatment with IV iron and rHuEPO did not accelerate the recovery from new-onset postoperative anemia among orthopedic surgery patients, besides achieving higher reticulocyte counts in the first week of surgery. No improvement in mobilization of storage iron was achieved with rHuEPO, and vitamin B_12_ supplementation is not required during the early postoperative period.

## Data Availability

The data that support the findings of this study are available from the corresponding author upon reasonable request. The data are not publicly available to protect patient privacy.
